# Safety and efficacy of volume-based feeding in critically ill, mechanically ventilated adults using the ‘Protein & Energy Requirements Fed for Every Critically ill patient every Time’ (PERFECT) protocol: a before-and-after study

**DOI:** 10.1186/s13054-019-2388-7

**Published:** 2019-04-02

**Authors:** Sue Brierley-Hobson, Graham Clarke, Vincent O’Keeffe

**Affiliations:** 1 Betsi Cadwaladr University Health Board, Bodelwyddan, LL18 5UJ UK; 2 Bangor University, Fron Heulog, Bangor, LL57 2EF UK

**Keywords:** Volume-based feeding, Critical care, Enteral, Protein, Mechanical ventilation

## Abstract

**Background:**

Underfeeding in critical illness is common and associated with poor outcomes. According to large prospective hospital studies, volume-based feeding (VBF) safely and effectively improves energy and protein delivery to critically ill patients compared to traditional rate-based feeding (RBF) and might improve patient outcomes. A before-and-after study was designed to evaluate the safety, efficacy and clinical outcomes associated with VBF compared to RBF in a single intensive care unit (ICU).

**Methods:**

The sample included consecutively admitted critically ill adults, mechanically ventilated for at least 72 h and fed enterally for a minimum of 48 h. The first cohort (*n* = 46) was fed using RBF, the second (*n* = 46) using VBF, and observed for 7 days, or until extubation or death. Statistical comparison of percentage feed volume, energy and protein delivered, plus indices of feed intolerance, were the primary outcomes of interest. Secondary observations included ventilation period, mortality, and length of ICU stay (LOICUS).

**Results:**

Groups were comparable in baseline clinical and demographic characteristics and nutrition practices. Volume delivered to the VBF group increased significantly by 11.2% (*p* ≤ 0.001), energy by 13.4% (*p* ≤ 0.001) and protein by 8.4% (*p* = 0.02), compared to the RBF group. In the VBF group, patients meeting > 90% of energy requirements increased significantly from 47.8 to 84.8% (*p* ≤ 0.001); those meeting > 90% of protein requirements changed from 56.5 to 73.9% (*p* = 0.134).

VBF did not increase symptoms of feed intolerance. Adjusted binomial logistic regression found each additional 1% of prescribed feed delivered decreased the odds of vomiting by 0.942 (5.8%), 95% CI [0.900–0.985], *p* = 0.010.

No differences in mortality or LOICUS were identified. Kaplan-Meier found a significantly increased extubation rate in patients receiving > 90% of protein requirements compared to those meeting < 80%, (*p* = 0.006). Adjusted Cox regression found the daily probability of being extubated tripled in patients receiving > 90% of their protein needs compared to the group receiving < 80%, hazard ratio 3.473, *p* = 0.021, 95% CI [1.205–10.014].

**Conclusion:**

VBF safely and effectively increased the delivery of energy and protein to critically ill patients. Increased protein delivery may improve extubation rate which has positive patient-centred and financial implications, warranting larger confirmatory trials. This investigation adds weight to the ICU literature supporting VBF, and the growing evidence which advocates for enhanced protein delivery to improve patient outcomes.

**Electronic supplementary material:**

The online version of this article (10.1186/s13054-019-2388-7) contains supplementary material, which is available to authorized users.

## Background

Critically ill patients are at high risk of morbidity, mortality and prolonged care needs [1]; implementing practical approaches to improve outcomes is of paramount importance [2]. During critical illness, evolutionary survival mechanisms release energy from stored body tissues to fuel life-supporting tasks [3]. The sacrifice from body stores is deleterious, and contributes to poor outcomes: when energy and protein is delivered to critically ill patients, this risk is ameliorated and recovery potential improved [1, 4, 5].

Despite the potential benefits of nutrition therapy, feeding is stopped intermittently in 85% of critically ill patients due to essential procedures and symptoms of feed intolerance [1]. These feed stops cause patients to meet only 40–60% of their energy and protein requirements, rather than allowing optimal delivery and meeting the minimum 80% recommended by clinical practice guidelines (CPGs) [1, 6].

Most intensive care units (ICUs) worldwide use an hourly ‘rate-based’ feeding (RBF) approach, without strategies to rectify feed deficits. Evidence suggests changing from a rate- to volume-based feeding (VBF) approach helps mitigate these accrued deficits without increasing feed intolerance in medical, and some surgical ICU patients [7–12], and by corollary, may improve clinical outcomes. Using the VBF approach, instead of prescribing an hourly feeding rate of, for example, 50 ml/h, a patient is prescribed 1200 ml/24-h period; systems are put in place to ensure the entire amount is delivered within 24 h.

CPGs encourage adoption of VBF in local ICU practice [1, 6]. The Heyland group [10–12], who named their VBF protocol: The *Enhanced* *Protein-Energy* *Provision* via *the Enteral Route in Critically Ill* *Patients* (PEPuP) protocol, openly share and encourage use of their resources to facilitate change in local ICUs, and endorse adapting their protocol to local context to facilitate implementation [13].

Not all studies show improved outcomes when energy and protein goals are achieved. The VBF studies by Haskins et al. [8], Taylor et al. [9] and Heyland et al. [11] reported no significant difference in LOS, mortality or ventilation was enabled by VBF. Some other studies evaluating overall nutritional delivery in the critically ill population [14–17] suggest reductions in mortality, ventilation period and LOS may be achieved when patients meet their energy and/or protein goals. Others warn that meeting ICU feeding targets triggers complex biochemical processes which have the opposite effect: increasing mortality, ventilation duration and LOS [18, 19], and recommend a less aggressive approach to feeding.

Three recent meta-analyses [20–22] found no difference in mortality, ventilation or hospital- or ICU-LOS when patients did or did not meet their energy or protein needs. The randomised controlled trials included in analyses used various strategies to improve nutritional delivery, such as faster feed starts and rate increase to ameliorate deficits [19], or managing gastric residual volumes or small bowel feeding. All meta-analyses found patients met sub-optimal levels of protein, which may be important. Emerging evidence suggests that with an increased turnover of up to 80% in critical illness, ICU patients have a much larger protein need than previously accepted [23–27]. It is common for protein to be targeted secondary to total energy in this patient group and is considered a neglected area of research [28, 29].

Deciding the optimal dose and timing of meeting the energy and protein needs of ICU patients remains controversial and subject to the impact of variable influences [30]. ASPEN [1] advise assessing for malnutrition risk, using either the Nutritional Risk Screening (NRS) 2002 or the Nutrition Risk in the Critically ill (NUTRIC) scores. NUTRIC was developed by Heyland et al. [31] to identify which ICU patients benefit most from nutritional support. The score has been externally validated in large prospective observational trials and found to identify the sickest patients more likely to have increased morbidity and mortality [32, 33]. Evidence suggests the NUTRIC score predicts energy and protein deficits in critically ill patients, but NRS does not [34], and that energy- and protein-related improvements in mortality are greatest in those patients with longer stay, and at highest risk calculated using NUTRIC [33, 35].

In January 2016, a Local Health Board (LHB) medical-surgical ICU multidisciplinary team commenced a prospective before-and-after study designed to compare nutritional delivery between RBF and VBF. The study was designed with the primary aim of confirming the hypotheses: that changing from RBF to VBF would significantly increase the percentage of prescribed feed volume, energy and protein delivered to adult critically ill patients, without altering feed tolerance. Secondary observations of interest included between-group comparisons of patients’ outcomes including mortality, length of ICU stay (LOICUS) and mechanical ventilation.

## Methods

### Permissions to undertake study

The study did not require informed patient consent: the system-level quality improvement initiative was designed to undertake a minimal-risk change in feed process which did not exceed the boundaries of standard clinical care, and could not take place practically if prior consent were required [11, 36]. The LHB ‘Research and Development’ department consented to the work as a service evaluation project without need to pursue ethical review. The required University Healthcare Sciences and Medical Sciences Academics Ethics Committee approval was obtained before data analysis.

### ICU characteristics

The adult, medical-surgical ICU is within a district general teaching hospital comprising 600–700 beds. Staffing is provided in a one-to-one nurse-to-patient ratio, and patients are overseen by a Consultant Intensivist. In the years spanning 2012–2015, quarterly ICU admissions were consistent at 181–204 patients, and average length of mechanical ventilation was 3.7–4.0 days [37].

### Recruitment

Data collection was undertaken prospectively in consecutively admitted, adult (≥ 18 years) patients who were mechanically ventilated for 72 h or more and fed for at least 48 h. The Local Health Board intensive care unit (LHB-ICU) does not currently use NUTRIC, and the 72-h duration was selected a priori as a method of sample restriction to define a level of disease acuity and longer stay.

Enteral feeding was commenced within 24 h of ventilation in stable patients. Only patients deemed clinically appropriate to receive full feeding by the medical or surgical team were included. VBF was undertaken from day 2 onwards, or once a patient was considered suitable to meet full-volume feeds. Patients initially nil by mouth, prescribed trophic feeding, or fed cautiously due to a risk of refeeding syndrome, were included if they were able to progress to full feeding within 72 h of ventilation.

Data was collected for up to 7 days, cessation of mechanical ventilation, death or ICU discharge, whichever occurred first.

Patients were excluded if they were pregnant, and/or were receiving parenteral or oral nutrition to limit the confounding effect of alternative nutritional support [5].

### Energy and protein requirements

The following principles were adhered to throughout the RBF and VBF periods.

Outside the dietitian’s working hours, the ICU used a ‘starter feeding regimen’ devised to closely meet the American Society for Parenteral and Enteral Nutrition (ASPEN) [1] energy and protein recommendations. It used a high protein, 1 kcal/ml feed containing 6.26 g of protein/100 ml for most patients (Osmolite HP [38]), or an isocaloric, lower protein, renal-conserving [39] feed for patients with established chronic kidney disease (Osmolite). The dietitian changed the feed prescription if required following assessment, using ASPEN [1] or other relevant guidelines [39, 40], and prescribing additional protein supplements when indicated (Additional file 1: Table S1).

Feed was progressed to target rate within 6 h of starting feed, unless prescribed trophic feeding, or considered at risk of refeeding syndrome, when feed targets were met gradually [1].

### The ‘PERFECT’ feeding protocol

The VBF protocol was adapted from PEPuP [13] and entitled: *Protein & Energy Requirements Fed for Every Critically ill patient every Time* (PERFECT); unlike PEPuP, baseline semi-elemental feeds, protein supplements and prophylactic prokinetics were not used.

The PERFECT toolkit instructed nurses how to increase feed rates, (maximum 150 ml/h) to compensate for feed-stops, and return to the initial goal rate at the beginning of the ICU 24 h period. For example, a patient prescribed 1200 ml would commence feeding at 50 ml/h at 0800 h; if the patient’s feed was off for 8 h, they had received 400 ml of feed prior, and there remained 8 h in the day on recommencing feeding, the deficit 800 ml (1200–400 = 800) would be given over 8 h at 100 ml/h (800/8). The 50 ml/h rate would recommence at 0800 h. A single end-of-day feed bolus up to 200 ml was given to replace remaining deficits. Boluses were not administered to jejunally fed patients.

Patients’ heads were elevated to 30–45° to reduce aspiration risk, and gastric-residual volume (GRV) was checked every 4–6 h. The ICU accepts and replaces GRVs up to 500 ml, with no change in feeding rate in the absence of other signs of intolerance.

Ward education was delivered by nurse-champions and the dietitian throughout December 2016 at daily and weekly team meetings. ‘How to’ booklets were kept at each bedside. One-to-one education and feedback was provided at the bedside, and continued ad hoc as required. Nursing daily documentation charts included an area to document feed deficits and corrections.

### Data collection

Baseline RBF data was collected prospectively for 7 months from April 2016. PERFECT was implemented January 2017, and data again collected prospectively in consecutive admissions for 6 months.

Data included age, gender, weight, height and body mass index (BMI) in kg/m^2^; ideal body weight (IBW) if obese, daily energy and protein requirements, the calories and grammes of protein prescribed per kilogramme, hours without feed, and the feed-volume prescribed and delivered in millilitres. The mean daily percentage of prescribed feed-volume, protein and energy delivered (including energy from propofol) was calculated for each patient, based on minimum requirement. Each patient’s mean daily kilocalories per kilogramme and grammes of protein per kilogramme delivered were noted. A whole day of ‘0’ energy and protein delivery was included as 0% achieved.

Total episodes of witnessed vomiting (gastric contents external to mouth) and regurgitation (gastric contents within the mouth) were noted; the expression ‘vomit’ hereon includes both. Mean daily episodes for patients who vomited were calculated. Patients with diarrhoea were noted. Three or more daily liquid stools were classified as diarrhoea using the World Health Organization definition [41], based on nurse perception of type 6–7 stools using the Bristol Stool Chart [42].

Daily patient GRV (millilitres) and the amount replaced were recorded. Prior to commencing VBF, the ICU changed from using 8-French (Fr) and 10-Fr NGTs (used in the RBF period) to using 12-Fr tubes, which withdraw substantially more GRV [43, 44], making the planned between-group comparison of aspirated volumes meaningless. Prokinetic prescription and the mean percentage GRV withdrawn and replaced per patient was compared between groups.

Subgroups of patients meeting < 80%, 80–89.9% and ≥ 90% of prescribed energy or protein were prepared for comparison, to explore any differences in clinical outcomes when patients achieved ‘over’ 80% of the ASPEN guideline recommendations, or specifically exceeded this.

Mean daily morning blood glucose and insulin requirement (mmol/L) per patient was noted, plus diabetes in past history as relevant to the frequency of hyperglycaemia [9].

Clinical measures recorded from the Case Mix Program Database (coordinated by the Intensive Care National Audit & Research Centre) [45] included the Acute Physiology and Chronic Health Evaluation II (APACHE-II) severity of illness score, advanced mechanical ventilation (days), ICU and 60-day hospital mortality (days) and length of ICU stay (LOICUS) (days: calculated from day 1 of ICU admission to when ‘ready for ICU discharge’ to account for delays in discharge); ICU admission diagnoses were summarised by surgery, respiratory, cardiovascular and ‘other’ (pancreatitis, gastrointestinal, neurological, sepsis, trauma, metabolic and haematological).

### Statistical analysis

#### Power analysis

The primary outcomes of interest were energy and protein delivery and feed tolerance, while secondary observations of interest included ICU and 60-day mortality, ventilation period and LOICUS.

For the primary outcomes of interest, the improvements seen in protein and energy delivered to patients in the published VBF studies were classified as a medium-to-large effect size (0.70) for energy, and small-to-medium effect size (0.4) for protein. The G*Power 3 Power Analysis Program [46], version 3.1.9.2, was used to conduct a priori analysis for one-tailed *t* tests and Mann-Whitney *U* using the estimated effect sizes, an *α*-error level of 0.05 and an 80% power. A minimum sample requirement of 37 patients per group was noted.

#### Data management

IBM SPSS version 22 (IBM Corp., USA, 2013) [47] was used in descriptive and inferential statistical tests unless otherwise stated. Statistical significance was accepted at the *α*-error level ≤ 0.05; post hoc significance levels are cited in reporting.

Categorical variables are reported as counts and percentages. These were analysed for differences in proportional frequency between RBF and VBF groups using *χ*^2^ (chi-square) Test of Homogeneity, Test of Two Proportions, or Fisher’s exact test when cell counts were less than 5. Continuous variables are described by their means and standard deviations (±) when normally distributed, or by medians and interquartile range (IQR) when non-normally distributed. Mean and median group differences were compared using independent two-sample *t* tests for normally distributed data, or Mann-Whitney *U* for non-normal distributions.

Effect sizes are reported for percentage differences in the volume, protein and/or energy delivered between the RBF and VBF groups.

Equivalence between the VBF and RBF groups for mean episodes of vomiting was explored [48, 49] using ‘two one-sided tests’ (TOST). NCSS version 11 (NCSS, LLC: USA, 2016) statistical analysis software was used with a pre-stated margin of equivalence of 20% of baseline [48]. Combining both patient groups, binomial logistic regression was used to predict the probability of vomiting, adjusted for daily mean GRV, percentage feed volume delivered, and group.

Secondary outcomes of interest: 60-day survival, discharge and extubation rate were subjected to Kaplan-Meier and Cox Regression.

Kaplan-Meier for 60-day hospital survival used ICU admission date as start; censoring was based on hospital discharge alive or up to 60 days in hospital alive. For extubation rate analysis, patients who died on the ICU were excluded; day 1 of intubation was the starting point; extubation up to and including day 10 was the event, with ventilated patients thereafter censored. For LOICUS, censoring was undertaken after day 14.

Cox regression was adjusted for APACHE-II, group, and the percentage of energy or protein delivered; the covariate diagnosis of ‘respiratory disease’ was added to extubation-rate analysis [50], and BMI 25–35 kg/m^2^/< 25 and > 35 kg/m^2^ to extubation-rate and mortality analyses.

#### Other

To identify predictors of increased mean morning blood glucose, insulin and propofol, multiple regression was adjusted for the percentage of prescribed energy delivered, APACHE-II, group, BMI and/or having diabetes.

## Results

### Patient characteristics

Both the RBF and VBF groups comprised 46 patients. There were no significant differences between the groups’ patient demographic, anthropometric and baseline clinical characteristics; other than patients in the VBF group were prescribed more propofol (288.9ml) compared to the RBF group (221.6ml) (*p =* 0.025), and there were more patients with a BMI 25–35 kg/m^2^ in the VBF group (65.2%) than in the RBF group (43.5%) (*p* = 0.036) (Table 1).

**Table 1:** Baseline demographic and clinical characteristics and nutrition practices

Variable	RBF (*n* = 46)	VBF (*n* = 46)	*p*
Age (years): mean (SD)	64.3 (± 14.5)	64.8 (± 13.4)	0.870
Female: *n* (%)	20 (43.5)	15 (32.6)	0.283
Male: *n* (%)	26 (56.5)	31 (67.4)	
Weight (kg): mean (SD)	81.8 (± 26.6)	84.3 (± 15.7)	0.598
BMI (kg/m^2^): median (IQR)	27.2 (22.2–31.7)	28.1 (24.6–32.4)	0.238
BMI 25–35 kg/m^2^: *n* (%)	20 (43.5)	30 (65.2)	0.036
BMI < 30 kg/m^2^: *n* (%)	33 (71.7)	28 (60.9)	0.270
BMI ≥ 30 kg/m^2^: *n* (%)	13 (28.3)	18 (39.1)	
Admission diagnosis: *n* (%)			0.379
◦ Respiratory	13 (28)	19 (41)	
◦ Cardiovascular	7 (15)	10 (22)	
◦ Surgery	12 (26)	8 (18)	
◦ Other	14 (31)	9 (19)	
APACHE-II: mean (SD)	18.0 (± 5.8)	17.2 (± 4.8)	0.458
Diabetes: *n* (%)	13 (28.3)	11 (23.9)	0.635
Daily propofol (ml): median (IQR)	221.6 (43.3–332.7)	288.9 (221.7–360.9)	0.025
Days on propofol: median (IQR)	4.0 (1.6–7.0)	4.0 (2.0–7.0)	0.375
≥ 1 feed stop: *n* (%)	37 (80)	35 (76)	0.613
Hours without feed: median (IQR)	488 6.5 (1.0–16.0)	414 4.0 (1–14)	0.379
Volume prescribed (ml/day): mean (SD)	1311.9 (± 273.3)	1322.6 (± 285.3)	0.843
Energy prescribed (kcal/day): mean (SD)	1755.4 (± 397.9)	1787.0 (± 283.1)	0.662
Protein prescribed (g/day): median (IQR)	85.0 (70.0–110.0)	90.5 (83.0–120.0)	0.062

*p* values found using *t* test for continuous normally distributed outcomes, Mann Whitney *U* for continuous non-normally distributed outcomes, and Test of Two Proportions or Test of Homogeneity for categorical variables

*n* number of patients, *SD* standard deviation, *IQR* interquartile range, *APACHE-II* Acute Physiology and Chronic Health Evaluation II, *BMI* body mass index, *RBF* rate-based feeding, *VBF* volume-based feeding, *ml* millilitres, *ml/day* millilitres per day, *kg* kilogrammes, *kcal/day* kcal per day, *g/day*: grammes per day

### Nutrition practices

**Fig. 1:**
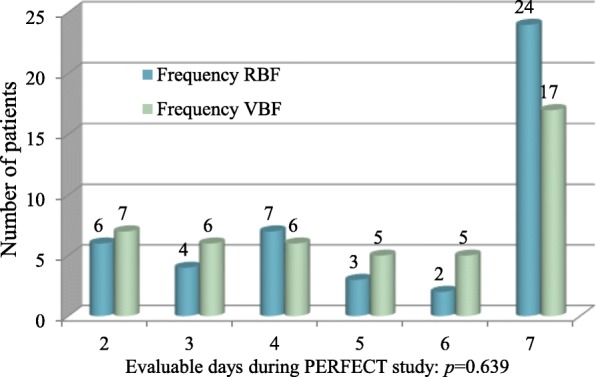
Number of evaluable feeding days in RBF and VBF groups. Figure shows the number of evaluable feeding days, and the *p* value of Fisher’s exact test demonstrating no significant group differences

All patients were fed by nasogastric tube, bar one in each group fed by nasojejunal tube. There was no difference in the number of evaluable feeding days (Fig. 1), *p* = 0.639, or duration without feed (*p* = 0.379) between groups (Table 1).

There was no significant difference in the volume of feed, total energy or protein prescribed to patients in the RBF and VBF groups (Table 1). Mean daily feed volume prescribed was 1312 ml (± 273 ml) in the RBF group, and 1323 ml (± 285 ml) in the VBF group, *p* = 0.843. Patients in the RBF group were prescribed a mean 1755 kcal/day (± 398 kcal/day) and the VBF group 1787 kcal/day (± 283 kcal/day) *p* = 0.662. Daily protein prescription in the RBF group was 85.0 g/day (IQR 70.0–110.0) and 90.5 g/day (IQR 83–120) in the VBF group, *p* = 0.062.

**Fig. 2:**
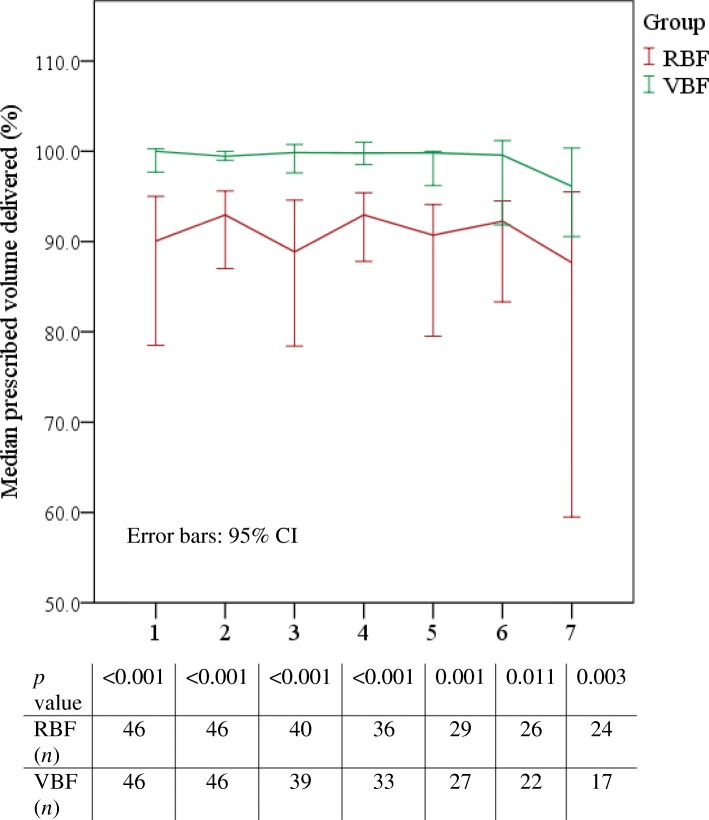
Daily median percentage feed volume delivered to RBF and VBF groups. Figure shows median 95% CI error bars, *n* per sample, and Mann-Whitney *U* result which found statistically significant increases in volume delivered to the VBF group compared to the RBF group every day

Prescribed feed volume delivered increased by 11.2% (Table 2) from a median 87.4% in the VBF group to 98.2% in the VBF group, *p* ≤ 0.001; *r* = 0.7 indicated a large effect size. The difference in feed volume delivered was significantly increased in the VBF group compared to the RBF group every day of the evaluable feeding period (Fig. 2). Feed volume delivered also increased significantly by 16.6% in surgical patients receiving VBF (*n* = 8) compared to those receiving RBF (*n* = 12), *p* = 0.001.

**Table 2:** Difference in feed volume, energy and protein delivered between the RBF and VBF groups

Variable	RBF*	VBF*	Change	*p*
Daily volume (ml): mean (SD)	1093.6 (± 285.3)	1290.7 (± 245.2)	> 197.2ml	0.001
% volume (all): median (IQR)	87.4 (80.5–92.1)	98.2 (95.4–100.0)	> 11.2%	< 0.001
% volume (surgical): Median (IQR)	82.8 (59.1–88.6) (*n* = 12)	99.4 (95.1–102.6) (*n* = 8)	> 16.6%	0.001
Energy (kcal/day)^a^: mean (SD)	1540.6 (± 380.2)	1784.5 (± 245.1)	> 243.9	< 0.001
% energy (all)^a^: mean (SD)	87.9 (± 13.8)	101.3 (± 11.7)	> 13.4	< 0.001
% energy (surgical)^a^: Median (IQR)	85.3 (67.2–93.4) (*n* = 12)	100.2 (93.4–112.3) (*n* = 8)	> 14.9	0.025
Daily kcal/kg^a,b^: mean (SD)	19.9 (± 4.9)	21.7 (± 3.8)	> 1.8	0.052
*° BMI < 30 kg/m*^*2*^ Mean (SD)	*n = 33* 22.1 (± 3.4)	*n = 28* 24.5 (± 2.1)	> 2.2	0.004
*°* BMI ≥ 30 kg/m^2^ Mean (SD)	*n = 13* 14.3 (± 3.5)	*n = 18* 17.6 (± 1.8)	> 3.4	0.006
Protein (g/day): mean (SD)	78.1 (± 19.4)	98.1 (± 23.7)	> 20	< 0.001
% protein (all): mean (SD)	89.2 (± 19.5)	97.6 (± 14.8)	> 8.6	0.022
% protein (surgical): Median (IQR)	88.3 (63.4–106.1)	108.1 (91.6–116.2)	> 19.8	0.031
Daily g/kg^c^: mean (SD)	1.2 (± 0.2)	1.4 (± 0.3)	0.2	< 0.001
*°* BMI < 30 kg/m^2c,e^ Median (IQR)	*n = 32* 1.2 (1.1–1.2)	*n = 26* 1.3 (1.1–1.3)	> 0.1	0.040
*°* BMI ≥ 30 kg/m^2d,e^ Median (IQR)	*n = 10* 1.2 (1.0–1.4);	*n = 15* 1.8 (1.5–1.9)	> 0.5	0.001
*°* Surgical Median (IQR)	*n* = 12 1.1 (0.8–1.3)	*n* = 8 1.3 (1.1–1.5)	> 0.2	0.069
*°* CKD: Mean (*n*)	1.0 (4)	1.0 (5)	0	

*p* values found using *t* test for continuous normally distributed outcomes, and Mann Whitney *U* for continuous non-normally distributed outcomes

*n* number of, *IQR* interquartile range, *SD* standard deviation, *BMI* body mass index, *RBF* rate-based feeding, *VBF* volume-based feeding, *ml* millilitres, *kg* kilogrammes, *kcal/day* kcal per day, *g/day* grammes per day, *kcal/kg* kcal per kilogramme, *CKD* chronic kidney disease

^a^kcal from feed and propofol

^b^kcal/kg of actual bodyweight

^c^g/kg of actual bodyweight

^d^g/kg of ideal bodyweight

^e^Excluding renal patients

*n = 46 unless otherwise stated

The percentage of prescribed energy (Table 2) delivered increased significantly by 13.4% in the VBF compared to the RBF group, from 87.9% (± 13.8%) to 101.3% (± 11.7%), 95% CI [8.1–18.7], *p* ≤ 0.001; eta^2^ = 0.22 indicated this was a large difference. Patients in the VBF group received more daily energy (1785 kcal/day, ± 245 kcal/day) than the RBF group (1541 kcal/day; ± 380 kcal/day), 95% CI [111.1–376.6], *p* ≤ 0.001.

The proportion of patients receiving > 90% of their energy prescription increased significantly from 47.8% in the RBF group to 84.8% in the VBF group (*p* ≤ 0.001) (Additional file 1: Table S2).

### Protein delivery

Prescribed protein delivered (Table 2) increased significantly by 8.4% (*p* = 0.02), by a mean 20 g per day, 95% CI [11.0–29.0], (*p* ≤ 0.001): from 89.2% (± 19.5%) in the RBF group to 97.6% (± 14.8%) in the VBF group, 95% CI [1.2–15.6], *p* = 0.02; a moderately sized difference according to the effect size eta^2^ = 0.06. Subgroup analysis found patients receiving over 90% of prescribed protein increased from 56.5% in the RBF group to 73.9% in the VBF group, *p* = 0.134 (Additional file 1: Table S3).

### Feed tolerance

There was no significant difference in patients experiencing at least 1 day of diarrhoea, with 26 in the RBF group (56.5%), and 18 (39.1%) in the VBF group, *p* = 0.095 (Table 3).

**Table 3:** Summary of descriptive and inferential analysis for feed tolerance

Variable	RBF (*n* = 46)	VBF (*n* = 46)	*p*
Patients with diarrhoea: *n* (%)	26 (56.5)	18 (39.1)	0.095
Mean GRV (ml): mean (SD)	37.8 (68.3)	153.5 (240.0)	See narrative
% GRV replaced: median (IQR)	100 (20–100)	100 (61–100)	0.621
Patients vomiting: *n* (%)	11 (23.9)	9 (19.6)	0.613
Prescribed prokinetics: *n* (%)	13 (28.3)	14 (30.4)	0.819
Insulin (U): median (IQR)	6.7 (0.0–38.7)	24.3 (0.0–39.7)	0.248
Mean morning blood glucose (mmol/L): median (IQR)	8.0 (7.1–8.8)	8.5 (7.8–9.5)	0.034

*n* number of, *IQR* interquartile range, *SD* standard deviation, *RBF* rate-based feeding, *VBF* volume-based feeding, *ml* millilitres, *U* units, *mmol/L* millimoles per litre

The GRV replaced in the RBF (100.0%) and VBF groups (100.0%) *p* = 0.521 were similar, and there was a non-significant difference in prokinetic prescription, with 28% of patients in the RBF group and 30% in the VBF group prescribed prokinetics, *p* = 0.819 (Table 3).

A Mann-Whitney *U* TOST identified episodes of vomiting reduced by over 20% in the VBF group (Additional file 1: Table S4). The binomial logistic regression adjusted odds ratio (Additional file 1: Table S5) found that for each additional 1% of prescribed feed delivered, there was a 0.942 (5.8%), 95% CI [0.900–0.985], *p* = 0.010 decreased odds of vomiting.

Mean morning BG (Table 3) was significantly higher in the VBF group (8.5 mmol/L) than the RBF group (8 .0 mmol/L), *p* = 0.034. Adjusted multiple regression found diabetes to be the only predictor of increased mean morning blood glucose, being 1.05 mmol/L greater in people with diabetes, than those without, 95% CI [0.456–1.646], *p* = 0.001 (Table 4).

**Table 4:** Multiple regression analysis: showing analysis of morning BG, plus insulin and propofol requirement

Variable and adjustment	Morning blood glucose			Insulin prescription			Propofol		
	*B*	*p*	95% CI	*B*	*p*	95% CI	*B*	*p*	95% CI
Group*	0.426	0.157	− 0.166 to 1.018	2.990	0.574	− 7.561 to 13.540	52.364	0.107	− 11.572 to 116.299
BMI	0.005	0.777	− 0.031 to 0.041	0.314	0.330	− 0.324 to 0.952	3.258	0.102	− 0.664 to 7.181
% Energy	0.011	0.316	− 0.010 to 0.032	0.203	0.272	− 0.162 to 0.568	1.197	0.294	− 1.058 to 3.451
APACHE-II	0.000	0.992	− 0.050 to 0.051	1.424	0.003	0.514 to 2.234	0.235	0.932	− 5.192 to 5.661
Diabetes^+^	1.051	0.001	0.456 to 1.646	40.088	< 0.001	29.013 to 51.163	–	–	–

*B* unstandardized regression coefficients, *APACHE-II* Acute Physiology and Chronic Health Evaluation II, *BMI* body mass index, *CI* confidence interval

*VBF versus RBF

^**+**^Having diabetes versus not

There was no significant difference in the mean insulin units prescribed to patients between the RBF (median 6.7; IQR 0.0–38.7) and VBF (median 24.3; 0.0–39.7) groups, *p* = 0.248. Though not significantly different in distribution [51], the medians were notably dissimilar (Table 3). Adjusted multiple regression found mean daily insulin prescription was predicted to be 40.1 units greater in people with diabetes than those without, 95% CI, [29.013–51.163], *p* ≤ 0.001, and for each increase in APACHE-II score, insulin prescription was predicted to increase by 1.4 units per day, 95% CI [0.514–2.234], *p* = 0.003.

None of the variables, specifically group, BMI, percentage energy delivered or APACHE-II score used in adjusted multiple regression, predicted propofol prescription (Table 4).

### Clinical outcomes

#### Mortality

**Fig. 3:**
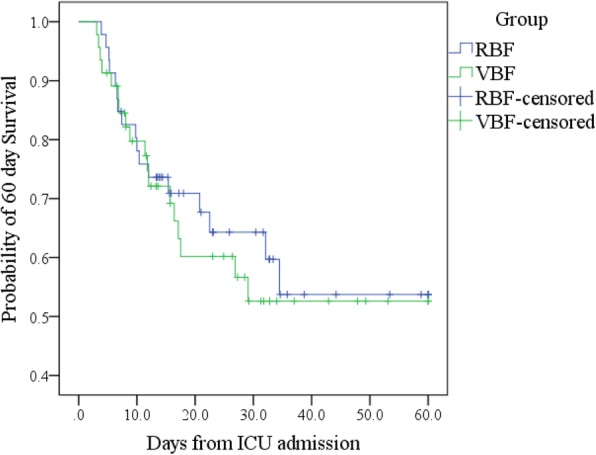
Kaplan-Meier 60-day survival curves by group; shows no difference in 60-day survival

Total ICU and hospital deaths were the same or similar in each group (Additional file 1: Table S6). Kaplan-Meier (Additional file 1: Table S6; Fig. 3) and log-rank test found no significant difference in survival distribution between groups (*p* = 0.693). Adjusted Cox regression (Additional file 1: Table S7) found neither the percentage of prescribed energy or protein delivered nor group or BMI range predicted survival time.

#### Mechanical ventilation

**Fig. 4:**
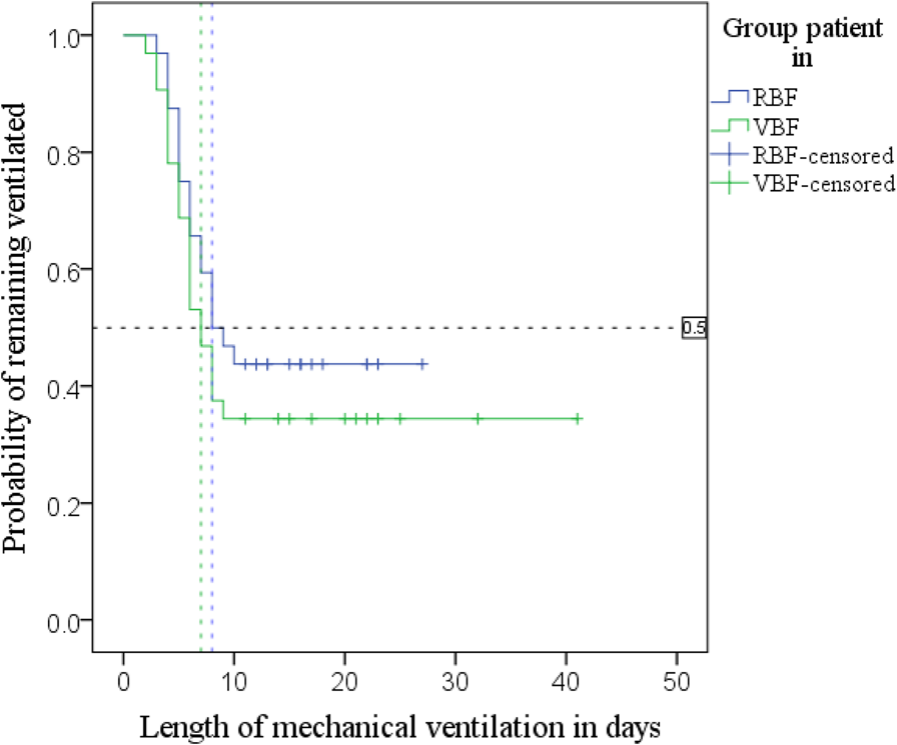
Kaplan-Meier curves; time to extubation by group

Kaplan-Meier (Fig. 4; Additional file 1: Table S6) with log-rank test found patients in the RBF group had a median time to extubation of 8 days, 95% CI [4.7–11.3], and the VBF group had a median time of 7 days, 95% CI [5.3–8.7]; *p* = 0.342.

Adjusted Cox regression (Additional file 1: Table S7) found that each 1% additional protein delivery increased the daily probability of extubation 1.022-fold (by 2.2%), *p* = 0.040, 95% CI [1.001–1.043]. The surviving 64 patients (minus one extreme outlier) were grouped by percentage of prescribed protein delivered: < 80%, 80–89.9% and ≥ 90%. A further adjusted Cox regression (Additional file 1: Table S7) found patients receiving 80–89.9% of prescribed protein did not have a significantly different time to extubation compared to those meeting < 80%, hazard ratio (HR) 1.635, *p* = 0.498, 95% CI [0.395–6.772]; however, the daily probability of being extubated more than tripled in patients receiving > 90% of their protein needs compared to the group receiving < 80%, HR 3.473, *p* = 0.021, 95% CI [1.205–10.014].

**Fig. 5:**
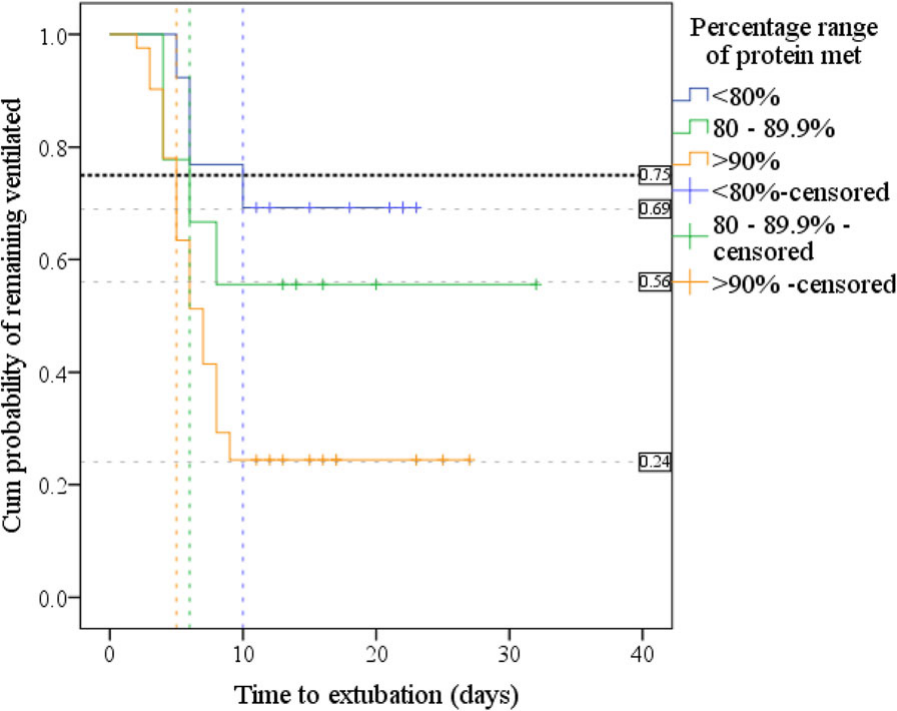
Kaplan-Meier curves for time to extubation by percentage of prescribed protein delivered: shows 75th centile, days by which 25% of each group extubated and the cumulative probability (discussed as %) of remaining ventilated at day 10

Another Kaplan-Meier was run using the same subgroups of percentage prescribed protein delivered. Kaplan-Meier curves (Fig. 5) suggested a substantially increased extubation rate in patients receiving > 90% of their protein needs; the cumulative probability of this group remaining ventilated at day 10 was 24%; probability was 56% in the group meeting 80–89.9%, and 69% in the patients who met < 80% of their protein requirements.

Log-rank test found significant differences in the distribution of extubation time between the 3 protein ranges, *p* = 0.012. Pairwise log-rank comparisons (Additional file 1: Table S8) were undertaken to compare ventilation distributions. Using a Bonferroni correction, with significance accepted at the *p* < 0.0167 level, there was a significant difference in ventilation distribution between the groups of patients receiving < 80% and ≥ 90% protein, *p* = 0.006.

The 75th centile is shown in Fig. 5 and Additional file 1: Table S8 and demonstrates the time by which 25% of patients were extubated: 25% of those receiving < 80% of protein were extubated by day 10, 95% CI [4.6–15.4], those receiving 80–89.9% by day 6, (SE not calculated), and > 90% by day 5, 95% CI [3.9–5.9].

#### Length of ICU stay

**Fig. 6:**
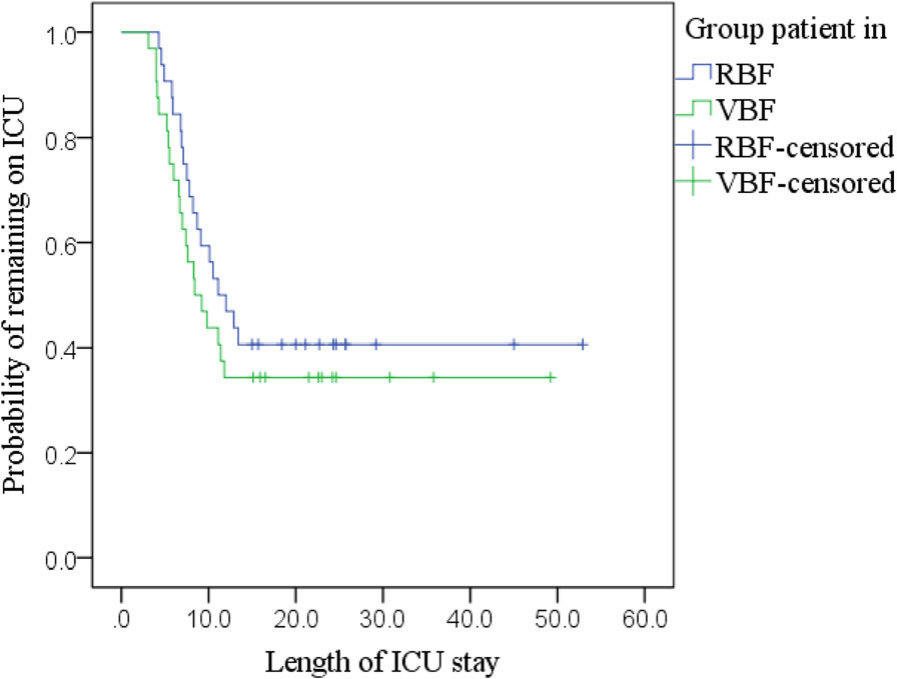
Kaplan-Meier curves: LOICUS by RBF and VBF group

Kaplan-Meier (Fig. 6; Additional file 1: Table S6) with a log-rank test found no statistically significant difference in the LOICUS between groups, *χ*^2^ (1) = 0.815, *p* = 0.367. Adjusted Cox regression identified no significant predictors of discharge rate (Additional file 1: Table S7).

## Discussion

This prospective before-and-after study suggests volume-based feeding safely improved patients’ feed volume, energy and protein delivery, and that increasing protein delivery increased the rate of extubation, but these factors did not influence mortality or LOICUS.

### Changes to delivery of feed volume, energy and protein

The percentage energy increase in the VBF group compares similarly to the VBF studies in other medical-surgical ICUs which improved delivery by 9.1–17% [8, 10–12]. The 8.6% protein increase achieved in the PERFECT study was exceeded in the Heyland et al. [10–12] PEPuP studies, with their improvements ranging from 11.3 to 14%, which is explained by the broad prescription of 24-g protein modules daily to patients, which were not used in the PERFECT study. In the PERFECT study, the increase in energy and protein delivery in the VBF group meant that patients in this group more closely met the ASPEN nutrition guideline recommendations targeted by the LHB-ICU, albeit protein targets were still not achieved in the cohort of patients with a BMI > 30 kg/m^2^. The achievement of 1.3 g of protein per kilogramme bodyweight in patients with a BMI < 29.9kg/m^2^ meets the minimum prescription recommended by the newly published European Society of Parenteral and Enteral Nutrition (ESPEN) ICU nutrition guidelines [52].

Like Taylor et al. [9], the small subgroup of surgical patients in the PERFECT study’s VBF group received significantly more energy and protein than the RBF group. These findings are unlike the surgical ICU study by Declercq et al. [7], who found no difference in energy or protein delivery using VBF, which was attributed to poor protocol compliance. Heyland et al. [11, 12] acknowledge that simply using a protocol may be insufficient to overcome individual unit cultural and systemic barriers. The PERFECT study utilised various system-level improvement techniques to facilitate adoption, such as identified project leadership, team coproduction, local adaptation, and staff education [53, 54]. Notably, the PERFECT study’s RBF group met over 85% of prescribed energy and protein needs, demonstrating the existing positive attitude to nutrition practice in the LHB-ICU, which likely fostered readiness to adopt the new approach.

### Feed tolerance

There was no difference between the RBF and VBF groups in patients experiencing diarrhoea. Taylor et al. [9] reported the number of patients with diarrhoea significantly increased in their VBF group, which was attributed to large (400 ml) intragastric feed boluses not used in the PERFECT study.

Analysis found that each additional 1% of feed volume delivered was associated with a significantly decreased odds of vomiting. This is physiologically conceivable given normal gastric retention and emptying processes [43, 55] and is supported by others’ work [9, 10, 56, 57].

Neither mean morning blood glucose (BG) levels nor insulin prescription was predicted by increased energy delivery or being in either group, and BG levels were maintained under 10 mmol/L as per current recommendations [1]. Obesity is independently characterised by insulin-resistance, which can be exacerbated by the added metabolic complexities of critical illness [58, 59]. Although benefits remain to be confirmed [52, 59], hypocaloric, high protein feeding is proposed to minimise the metabolic consequences of over-feeding in obese patients, plus preserve nitrogen balance and lean mass [52, 58–60]. This approach is recommended by ASPEN [1] and was used in the PERFECT study for this patient cohort. Managing the energy requirements of overweight critically ill patients has not yet been addressed by research [52]. We note with interest that ESPEN [52] have very recently recommended a more conservative approach to prescribing energy in patients with a BMI > 25 kg/m^2^. In the PERFECT study, BMI did not predict elevated blood glucose or insulin requirements, suggesting our relatively conservative approach to energy prescription mitigated risk.

### Clinical outcomes

#### Mortality

Unlike the PERFECT study which found feeding improvements did not change mortality outcomes, some studies suggest improved energy and/or protein delivery improves survival [15, 17]; others suggest outcomes are worse when patients meet these targets, and advise a less aggressive approach [18, 19].

An observational point-prevalence study of 2772 patients from 167 medical-surgical ICUs by Alberda et al. [4] found that 1000 kcal/day and 30 g protein delivered were associated with reduced 60-day mortality, though only in patients with a BMI < 25 kg/m^2^ and > 35 kcal/m^2^, who may be less susceptible to the earlier metabolic consequences of critical illness [1, 5]. In the PERFECT study, 43.5% of patients in the RBF group, and 65.2% in the VBF group, had a BMI of 25–35 kg/m^2^: these patients may not have benefitted from early macronutrient manipulation, substantially limiting the likelihood of statistically finding enhanced mortality outcomes in the small number of patients with a lower or higher BMI.

It may be that patients with a lower BMI, having less reserve, benefit more from supplementation, while heightened alterations to macronutrient utility in critically ill obese cohorts can make both over- and under-feeding detrimental [1, 58, 60, 61]. This may explain the increased mortality attributed to enhanced feeding suggested by studies such as Braunschweig et al. [19], who estimated and often exceeded energy requirements at 30 kcal/kg in a largely obese cohort. Using such estimations in the acute phase of ICU admission is associated with a metabolic burden which is not recommended [1, 58, 61, 62].

Some studies suggest ‘forced feeding’ in the first week of critical illness inhibits autophagy [63, 64]. The physiological process of autophagy regulates inflammation, clears toxic cell damage and supports protein synthesis in starvation [65]. Feeding, insulin and hyperglycaemia inhibit autophagy, prompting the hypothesis that autophagy is prevented when need is greatest in critical illness, leading to accelerated muscle loss.

Autophagy is influenced by the severity of oxidative stress [65], suggesting those with greater stress may be more vulnerable to autophagy inhibition caused by overfeeding. As characterised by their need for advanced mechanical ventilation, 89.1% of patients in the PERFECT study’s RBF group and 97.8% in the VBF group required propofol, and the VBF group received significantly more median units. Outcomes were not worse in the PERFECT study’s VBF group despite receiving more energy and propofol than the RBF group, challenging the theory of detrimental autophagy inhibition.

The role of autophagy in critical illness and feeding is poorly understood: McClave and Weijs [65, 66] argue evidence does not support underfeeding, though suggest preventing hyperglycaemia by avoiding excessive energy provision is a prudent approach to limit inhibition. They note ‘excessive’ does not equate to meeting the guideline-recommended energy and protein targets utilised in the PERFECT study, suggesting patients in the more ‘aggressively’ fed VBF group were protected by targeting low-to-moderate energy and higher-protein feeding in all recruits, which as summarised earlier did not cause hyperglycaemia.

#### Ventilation

Enhanced protein delivery significantly increased the rate of extubation of survivors, with the daily probability of being extubated more than tripling in the PERFECT study’s group of patients receiving > 90% of their protein needs compared to patients receiving < 80%. These findings were unlike Alberda et al. [4], who found each additional 30 g protein was not associated with more ventilator-free days (VFDs). Alberda et al. [4] only reached 60% of target protein equalling a mean 47 g/day compared to the PERFECT study’s often supplemented mean 78.1–98.1 g/day in the RBF and VBF groups; perhaps the Alberda et al. [4] study’s patients did not meet enough protein to see the ventilation improvement. This might also explain the lack of improvement seen by Heyland et al. [11] in their VBF study given that patients met only 48% of energy and protein from all sources.

Studies such as those undertaken by Alberda et al. [4] have been criticised for undertaking separate protein analyses when using fixed-ratio feeds without protein supplementation given their propensity to underfeed protein: while patients may receive ‘more’, it could be insufficient to change outcomes [29, 67, 68].

Three meta-analyses [20–22] found no difference in mortality, LOS, or ventilation when patients did or did not meet their energy and protein needs; however, none of the studies included in meta-analyses achieved more than a mean 1.1 g/kg of protein. This factor may distinguish the PERFECT study’s extubation findings from some others’ work. Metabolic studies suggest 1.5–2.5 g/kg protein/day is required for catabolic critically ill patients to reach muscle protein synthesis [28, 69]. Reduced muscle mass inevitably weakens function and has been linked to reduced respiratory power and prolonged ventilation [66, 69], so the ventilation improvement seen in the PERFECT study is plausible and compares to some others’ findings [5, 60].

Elke et al. [5] completed secondary analysis using pooled data from the Alberda et al. [4] and Heyland et al. [10] studies, to achieve a sample of 2270 patients, who, like the PERFECT study, were ventilated for over 72 h and exclusively enterally fed. Patients’ nutrition was split into tertiles of achievement and analysed in separate logistic and linear regression models for energy and protein, adjusted for age, BMI and APACHE-II. The study found each additional 1000 kcal/day and 30 g protein reduced 60-day mortality and increased VFDs. In sensitivity analysis of survivors fed for the first 7 days of admission, ventilation improvements only persisted for increased protein provision.

With a mean age 62 years, BMI 27.6 kg/m^2^, median ventilation of 8.4 days, mortality 31%, LOICUS 11.5 days and being predominantly male, Elke et al. [5] argue their study is reflective of typical ICU populations and is certainly comparable to the PERFECT study’s recruits.

Hoffer and Bistrian [28] suggest patients with an ICULOS longer than 3.8 days will particularly benefit from additional protein to alleviate muscle atrophy. This may be especially true for patients with pre-existing atrophy, such as malnourished and/or inactive elderly and obese patients who depend more on feeding given their reduced ability to achieve nitrogen-balance [28]. Patients in the PERFECT and Elke et al. [5] studies were included for a minimum of 3-day ventilation and largely fit this description.

#### Strengths and limitations

The strengths and limitations of the PERFECT study require consideration.

The sample of 46 patients per group exceeded the minimal sample requirement identified through power analysis, which gives confidence in the findings suggesting feed volume, energy and protein delivery was significantly increased in the VBF compared to the RBF group, without adverse effects.

Heyland et al. [12] questioned whether VBF improvements in feed delivery could be reproduced using polymeric, rather than semi-elemental feeds. The PERFECT study strongly suggests this is possible and, furthermore, safely achieved without prophylactic prokinetics as utilised by Heyland et al. [10–12]. The successfully improved nutritional delivery and safety seen adds detail to the published research about volume-based feeding.

Recruiting only patients ventilated for 72 or more hours and amenable to VBF within the observation period ensured the study represented patients with a prolonged ICU stay and had some uniformity of disease acuity [69]. The RBF and VBF groups were well-matched with regard to baseline clinical and demographic factors and nutrition practices.

Nevertheless, outcome findings require cautious interpretation. We acknowledge the confounding influence of illness severity and population heterogeneity challenges findings which suggest changes in outcomes are causally related to nutrition in ICU observational studies [70]. Clinical outcomes were considered observations of interest and were not subject to power analysis. Knowledge of which patients benefit from ICU energy and protein remains elusive, with some suggesting three groups likely; those who do, or do not recover regardless, and those who benefit, such as patients particularly susceptible to lean-tissue atrophy, and/or otherwise identified as high risk [28, 31, 71].

While discussion established this population might be reasonably represented in the PERFECT study, if the outcomes of many recruits occurred independently of energy and protein delivery, isolating nutrition’s treatment effect in this small sample would be very challenging, particularly when, although statistically significant, the absolute difference in energy and protein delivery between groups was relatively small. We recognise this is especially true of mortality, with much larger observational and randomised controlled trials, designed and powered specifically to measure this outcome, still yielding conflicting results [52].

The suggestion that increased protein delivery predicted the probability of earlier extubation in the PERFECT study is exciting, and links to the recent call to research by Hurt et al. [72] to explore protein-related improvements in short-term outcomes. The findings from the small sample were strengthened by use of a Bonferroni correction; nevertheless, this result must be interpreted with caution and, while encouraging, should only be used in future hypothesis development.

Adjustments for the most pertinent covariates in regression analyses were made, but, to optimise statistical quality, only a limited number were introduced; there may be other, unadjusted confounding influences.

As patients were not followed up once extubated, it cannot be assumed that energy and protein needs continued to be met, and inconstant intakes may have influenced the reported outcomes.

Finally, patients in the PERFECT study were not randomised, which was justified by the nature of the system-level, quality improvement intervention [73].

## Conclusion

The investigation found the PERFECT VBF feeding protocol significantly enhanced feed volume, energy and protein delivery to prolonged, mechanically ventilated patients in the LHB-ICU, without increasing feed intolerance. The exciting finding that enhanced protein delivery may improve ventilation is considered plausible, albeit requires further confirmatory study.

The approach is now embedded in daily practice on our ICU. As noted, patients in the PERFECT study were quite ‘typical’ of ICU admissions elsewhere, suggesting the findings will be useful to those working in similar medical-surgical ICUs considering adopting this approach. We recognise that our ICU patients met over 80% of protein and energy targets prior to commencing VBF: one might consider VBF unnecessary in such a cohort. As delineated in Fig. 2, using a VBF strategy significantly improved the likelihood of consistently achieving daily feed targets in all patients; therefore, we do consider this approach worthwhile in facilitating optimal energy and protein delivery regardless of baseline.

Large-scale research to demonstrate the safety and efficacy of VBF in other ICU populations has merit. That said, this analysis and the resulting discussion highlight that a unifying characteristic of many studies published thus far is a failure to optimise protein delivery: this may be key to improving ICU outcomes and suggests just doing ‘more of the same’ is not enough. Efforts at improving feeding in ICUs meeting low volumes are valuable, but these efforts should be aimed at increasing supplemented protein delivery, not just total energy.

The science which increasingly hints at patient groups more susceptible to the benefits of improved nutrient delivery, such as those achieving high NUTRIC scores, indicates a useful research target to trial VBF. Targeting such a group would help overcome the difficulty of ascribing credit to nutrition in improving outcomes in such a heterogeneous population. The evidence from NUTRIC studies so far does not show that feeding harms patients at low risk with short stays, and as it is often difficult to predict those who will have the longer ICU course, Compher et al. [35] endorse continuing to optimise feeding in all patients, which this investigation has shown, is enabled by the PERFECT feeding protocol in our ICU. As noted, we do not calculate the NUTRIC score at present; this will be a recommendation for our Unit so we can be prepared to respond to emerging findings.

To optimise interpretation and generalisability, large, multicentre randomised controlled trials must be designed to measure outcomes related to improved protein delivery, using adequately powered samples for pre-specified effect sizes [74], a priori determined patient outcomes, and subject to powerful statistical analysis.

## Additional file

Additional file 1: **Table S1.** Calculating patients’ energy and protein prescription (using ASPEN guidelines [1] unless otherwise stated). **Table S2.** χ^2^ test of homogeneity showing group frequency distributions for the percentage range of energy delivered. **Table S3.** χ^2^ test of homogeneity showing group frequency distributions for the percentage range of protein delivered. **Table S4.** Equivalence TOST: daily episodes of vomiting. **Table S5.** Binomial logistic regression predicting odds of vomiting for mean GRV, percentage feed delivered, and group. **Table S6.** Summary of analysis for ICU and hospital mortality, ventilation period and LOICUS by group. **Table S7.** Results of adjusted Cox regression for 60-day survival, length of ventilation and LOICUS. **Table S8.** Results of Kaplan-Meier ventilation period by percentage range of prescribed protein delivered: showing 75th quartile, total events and pairwise log-rank comparisons of ventilation distribution: (significance accepted at *p* < 0.0167). (DOCX 24 kb)

## Abbreviations

ABW: Actual bodyweight
APACHE-II: Acute Physiology and Chronic Health Evaluation II (score)
BG: Blood glucose
BMI: Body mass index
CI: Confidence interval
CKD: Chronic kidney disease
CPG: Clinical practice guidelines
CVVHDF: Continuous venovenous hemodiafiltration
Energy: Expressed as kilocalories or kcal
g: Grammes (when discussing grammes of protein)
g/kg: Grammes (of protein) per kilogramme
GRV: Gastric-residual volume
HR: Hazard ratio
IBW: Ideal bodyweight
ICNARC: Intensive Care National Audit & Research Centre
ICU: Intensive care unit
INTACT: Intensive nutrition in acute lung injury: a clinical trial
kcal: Kilocalories
kcal/day: Kilocalories per day
kcal/kg: Kilocalories per kilogramme
kg: Kilogrammes
LHB: Local Health Board
LHB-ICU: Local Health Board intensive care unit
LOICUS: Length of intensive care unit stay
LOS: Length of stay
*M*: Mean difference
ml: Millilitre
mmol/L: Millimoles per litre
*n*: Number of
NGT: Nasogastric tube
NHS: National Health Service
*p*: Value denoting statistical significance
PEPuP: Enhanced Protein-Energy Provision via the Enteral Route in Critically Ill Patients
PERFECT: Protein & Energy Requirements Fed to Every Critically ill patient every Time
RBF group: The group who received rate-based feeding
RBF: Rate-based feeding/process
SD: Standard deviation; the symbol ‘±’ is used throughout to represent mean standard deviation
*t*: *t* test statistic
*U*: Mann-Whitney *U* test statistic
VBF group: The group who received volume-based feeding
VBF: Volume-based feeding/process
*χ*^2^: Chi-square
