# Supplementary material for: Safety and efficacy of volume-based feeding in critically ill, mechanically ventilated adults using the ‘Protein & Energy Requirements Fed for Every Critically ill patient every Time’ (PERFECT) protocol: a before-and-after study
Source: Crit Care. 2019 Apr 2;23:105. doi: 10.1186/s13054-019-2388-7 (PMC6444687; doi:10.1186/s13054-019-2388-7)
Supplement: Supplementary file 1 — Table S1. Calculating patients’ energy and protein prescription (using ASPEN guidelines [1] unless otherwise stated). Table S2. χ2 test of homogeneity showing group frequency distributions for the percentage range of energy delivered. Table S3. χ2 test of homogeneity showing group frequency distributions for the percentage range of protein delivered. Table S4. Equivalence TOST: daily episodes of vomiting. Table S5. Binomial logistic regression predicting odds of vomiting for mean GRV, percentage feed delivered, and group. Table S6. Summary of analysis for ICU and hospital mortality, ventilation period and LOICUS by group. Table S7. Results of adjusted Cox regression for 60-day survival, length of ventilation and LOICUS. Table S8. Results of Kaplan-Meier ventilation period by percentage range of prescribed protein delivered: showing 75th quartile, total events and pairwise log-rank comparisons of ventilation distribution: (significance accepted at p < 0.0167). (DOCX 24 kb) [file 13054_2019_2388_MOESM1_ESM.docx]

Table S1 Calculating patients’ energy and protein prescription (using ASPEN guidelines [1] unless otherwise stated)

| **BMI (kg/m^2^)** | **Energy (kcal/kg/day)** |
| --- | --- |
| ≤29.9 | 25* |
| 30-50 | Range 14* [1] to basal metabolic rate [40] |
| >50 | 22-25^+^ |
|  |  |
| **BMI (kg/m^2^)** | **Protein (g/kg/day)** |
| ≤29.9 | 1.2-1.5* |
| ≥30 | 2-2.5^+^ |
| CKD without CVVHDF | 0.8–1g/kg^+^ [39] |

***Key:*** ** multiplied by actual bodyweight; ^+^ multiplied by ideal bodyweight; BMI: body mass index; g (or) kcal/kg/day: grams (or) kcal per kilogram per day; CKD: chronic kidney disease; CVVHDF: continuous venovenous hemodiafiltration.*

Table S2 X^2^ test of homogeneity showing group frequency distributions for the percentage range of energy delivered

| Energy delivered | RBF  *n* (%) | VBF  *n* (%) | Fisher’s exact *p* | Pairwise *p^*^* |
| --- | --- | --- | --- | --- |
| <80% | 7 (15.2) | 2 (4.3) | 0.001 | 0.158 |
| 80-89.9% | 17 (37.0) | 5 (10.9) |  | 0.006 |
| >90% | 22 (47.8) | 39 (84.8) |  | <0.001 |

***Key: ^*^****Statistical significance accepted at p <0.0167; n: number of; RBF: rate based feeding; VBF: volume-based feeding.*

Table S3 X^2^ test of homogeneity showing group frequency distributions for the percentage range of protein delivered

| Protein delivered | RBF *n* (%) | VBF *n* (%) | *P^*^* |
| --- | --- | --- | --- |
| <80% | 12 (26.1) | 5 (10.9) | 0.134 |
| 80-89.9% | 8 (17.4) | 7 (15.2) |  |
| >90% | 26 (56.5) | 34 (73.9) |  |

***Key:*** *^*^Bonferonni correction p* <0.0167; *n: number of; RBF: rate based feeding; VBF: volume-based feeding.*

Table S4 Equivalence TOST: daily episodes of vomiting

| Group | Median daily vomit episodes | 95% CI | *U* | | Mean rank | *z*  (-Margin <diff, diff <Margin) | Equivalence *p* |
| --- | --- | --- | --- | --- | --- | --- | --- |
|  |  |  | Lower | Upper |  |  |  |
| RBF (*n*=11) | 0.33 | 0.14-0.57 | 56 | 37 | 115.5 | -0.4957, 0.9533 | 0.310 |
| VBF (*n*=9) | 0.29 | 0.17-0.67 | 43 | 62 | 94.5 |  |  |

***Key****: n: number of; RBF: rate based feeding; VBF: volume-based feeding.*

Table S5 Binomial logistic regression predicting odds of vomiting for mean GRV, percentage feed delivered, and group

| Variable | *p* | Odds Ratio | 95% CI |
| --- | --- | --- | --- |
| **% Volume delivered** | **0.010** | **0.942** | **0.900-0.985** |
| Mean GRV | 0.488 | 1.001 | 0.998-1.004 |
| Group* | 0.430 | 1.677 | 0.464-6.062 |

***Key****: *VBF compared to RBF; CI: confidence interval.*

Table S6 Summary of analysis for ICU and hospital mortality, ventilation period and LOICUS by group

| **Mortality** | | | |
| --- | --- | --- | --- |
| **Variable** | **RBF** | **VBF** | ***p*** |
|  | ***n*=46** | ***n*=46** |  |
| **ICU mortality:** *n(a)* (%) | 14 (30.4) | 14 (30.4) | 1.000 |
| **Hospital mortality**: *n(a)* (%) | 17 (37.0) | 18 (39.1) | 0.830 |
| **60-day survival*:** mean days; (95% CI) | 39.6 (32.0-47.1) | 37.7 (30.0-45.5) | 0.693 |
| **Time to extubation & LOICUS (survivors)** | | | |
|  | ***n*=32** | ***n*=32** | ***p*** |
| **Extubation***: *n(b);* median days; (95% CI) | *18;* 8.0 (4.7-11.3) | *21;* 7.0 (5.3-8.7) | 0.342 |
| **LOICUS***:  *n(b);* Median days; (95% CI) | *19*; 11.1 (7.2-15.0) | *21*; 8.4 (5.4-11.5) | 0.367 |

***Key****: n: number in group; n(a): number (and percentage) of patients who died while on ITU or in hospital; n(b): number of uncensored patients experiencing event; RBF: rate based feeding; VBF: volume based feeding; CI: confidence interval; LOICUS: length of intensive care unit stay; p-values calculated using Test of Two Proportions; *Kaplan Meier with p value calculated using log-rank test.*

Table S7 Results of adjusted Cox regression for 60-day survival, length of ventilation and LOICUS

| Variable & adjustment | *60-day survival* | | *Length of Ventilation* | | *LOICUS* | |
| --- | --- | --- | --- | --- | --- | --- |
|  | HR (95% CI) | *p* | HR (95% CI) | *p* | HR (95% CI) | *p* |
| APACHE-II | 1.17 (1.09-1.26) | <0.001 | 1.03 (0.95-1.11) | 0.55 | 1.00 (0.92-1.08) | 0.95 |
| % Energy | 1.01 (0.98-1.04) | 0.66 | 0.99 (0.96-1.02) | 0.68 | 1.00 (0.98-1.03) | 0.85 |
| % Protein | 1.01 (0.99-1.03) | 0.60 | **1.02 (1.00-1.04)** | **0.04** | 1.02 (1.00-1.04) | 0.11 |
| Group*  BMI 25-35kg/m^2^** | 1.27 (0.57-2.87)  0.97 (0.46-2.05) | 0.56  0.94 | 1.24 (0.60-2.55)  0.60 (0.45-1.60) | 0.56  0.60 |  |  |
| Protein 80-89.9%**^+^** |  |  | 1.64 (0.40-6.77) | 0.50 |  |  |
| Protein ≥90%**^+^** |  |  | **3.47 (1.21-10.01)** | **0.02** |  |  |
| Respiratory |  |  | 0.80 (0.41-1.56) | 0.51 |  |  |

***Key****: *VBF compared to RBF group; **compared to BMI <25 / >35kg/m^2^;* ***^+^****compared to patients meeting <80% of prescribed protein; HR: Hazard ratio;*  *APACHE-II: Acute Physiology and Chronic Health Evaluation; BMI: Body mass index; RBF: rate based feeding; VBF: volume-based feeding; LOICUS: length of intensive care unit stay.*

Table S8 Results of Kaplan-Meier ventilation period by percentage range of prescribed protein delivered: showing 75^th^ quartile, total events and pairwise log-rank comparisons of ventilation distribution: (significance accepted at p < 0.0167)

| Protein | 75^th^ quartile (days) | 95% CI | Events (*n*) | Pairwise log-rank comparisons  *p* | |
| --- | --- | --- | --- | --- | --- |
|  |  |  |  | vs 80% | vs 89.9% |
| <80% | 10.0 | 4.563-15.437 | 4 |  | 0.443 |
| 80-89.9% | 6.0 | - | 4 | 0.443 |  |
| ≥90% | 5.0 | 3.900-6.100 | 31 | 0.006 | 0.120 |

***Key****: n = number of.*
